# Transcriptome sequencing data sets of *Escherichia coli* K-12 MG1655 treated with novobiocin, tetracycline, and rifampicin

**DOI:** 10.1128/mra.01169-24

**Published:** 2025-05-22

**Authors:** Alexander Balkin, Andrey Plotnikov, Tatiana Konnova, Elena Shagimardanova, Hamza Hamo, Yuri Gogolev, Natalia Gogoleva

**Affiliations:** 1 Kazan Institute of Biochemistry and Biophysics, Kazan Scientific Center of Russian Academy of Sciences, Kazan, Russia; 2 Institute for Cellular and Intracellular Symbiosis, Ural Branch of the Russian Academy of Sciences, Orenburg, Russia; 3 Center for Personalized Medicine, Loginov Moscow Clinical Scientific Center, Moscow, Russia; 4 Institute of Fundamental Medicine and Biology, Kazan Federal University 473102, Kazan, Russia; 5 Research Department for Limnology, Mondsee, Universität Innsbruck, Mondsee, Austria; Rochester Institute of Technology, Rochester, New York, USA

**Keywords:** *Escherichia coli* gene expression, antibiotic resistance, RNA-seq

## Abstract

This report presents transcriptome datasets for *Escherichia coli* MG1655 treated with novobiocin, rifampicin, and tetracycline. Clusters of genes that respond to inhibition of RNA synthesis, protein synthesis, and DNA gyrase activity have been identified. The data may help to understand the mechanisms of general and specific resistance in *E. coli*.

## ANNOUNCEMENT

*Escherichia coli* MG1655 is a model organism for which detailed genomic information is available and extensive transcriptomic analyses have been performed (1–4). Like many enterobacteria, *E. coli* is resistant to novobiocin (5, 6); however, transcriptomic data describing this resistance are not available. Using the Cappable-Seq method, we described the repertoire of active transcriptional start sites of MG1655 upon treatment with novobiocin, tetracycline, and rifampicin (7). Here, we present an RNA-seq data set for this strain under the same conditions. Taken together, these data provide a snapshot of transcriptional activity changes in *E. coli*, revealing the bacterial adaptive potential through specific and nonspecific responses to inhibitors of transcription, translation, and DNA gyrase activity, which will expand our understanding of bacterial resistance for clinical and environmental applications.

**Fig 1:**
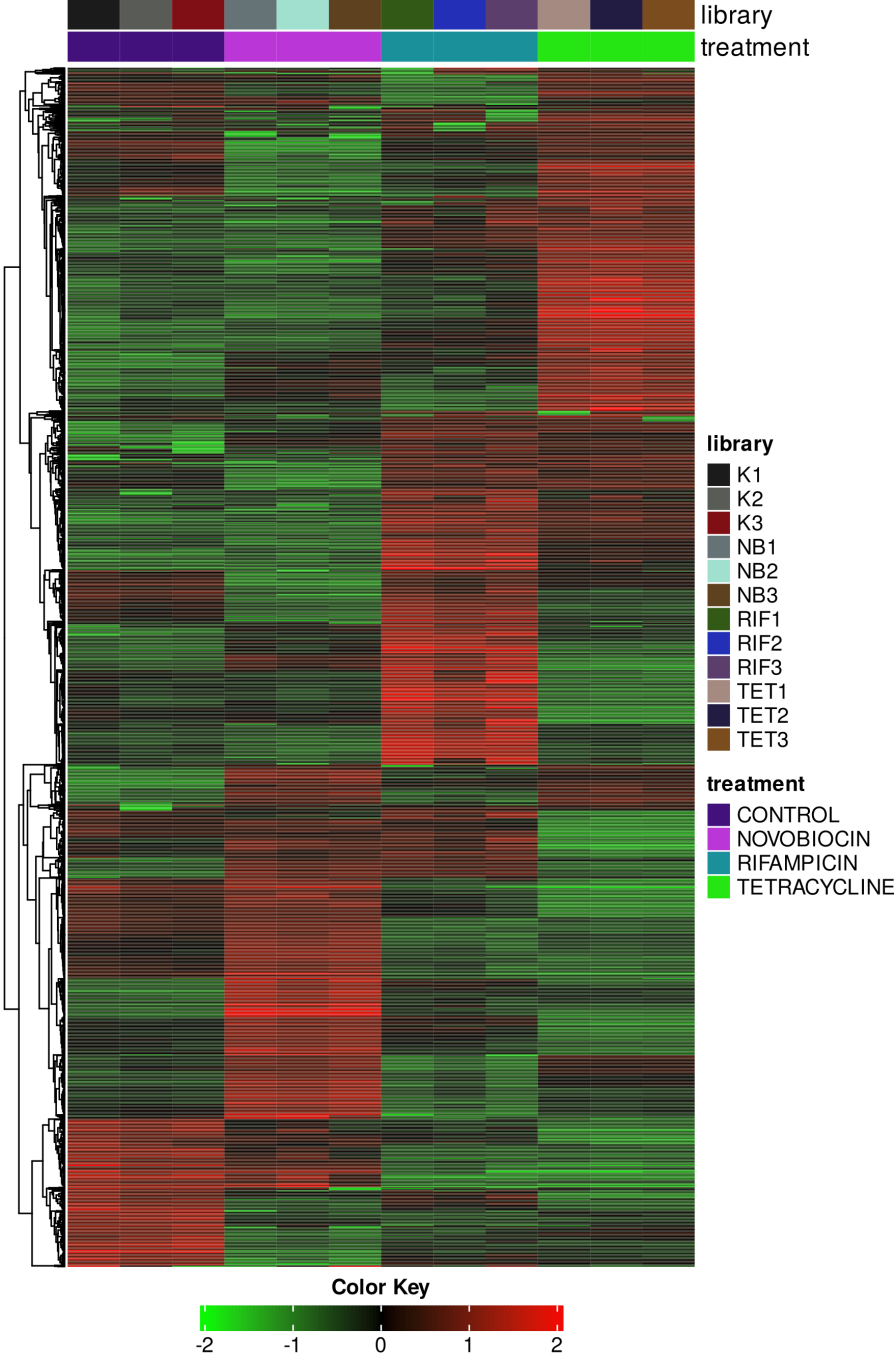
Heat map showing the Pearson hierarchical clustering of top 2,000 genes. Regularized log (Rlog) transformation was used for gene expression values. The data is centered by subtracting the average expression level for each gene. The color scale reflects the log2 relative transcriptional activity of the genes shown.

*E. coli* MG1655 cultures were grown in Luria-Bertani broth at 37°C with shaking (190 rpm). In the middle of the exponential phase (OD_600_ = 0.6), tetracycline, rifampicin, or novobiocin was added at four different concentrations. Concentrations that, after 1 h treatment, reduced the number of CFU by 50% compared to the control were selected for further experiment. The determined concentrations were 12.5, 50, and 100 mg/L for tetracycline, rifampicin, and novobiocin, respectively. Initial changes in the transcriptome were the focus of the experiment. Bacterial cells were fixed 15 min after treatment, when a divergence in the growth curves of the control and experimental cultures was observed, with an equal volume of 19% ethanol and 1% phenol, pH 5.5, on ice for 30 min. It has previously been shown that this fixation ensures reproducible quality of RNA samples (7). Bacterial RNA was isolated using TRIzol reagent and TURBO DNA-free kit (Thermo Fisher Scientific, Waltham, MA, USA). Ribosomal RNA was depleted using the Illumina Ribo-Zero Plus rRNA Depletion Kit (Illumina, San Diego, CA, USA). Libraries were generated using the NEBNext Ultra II Directional RNA Library Prep Kit for Illumina (New England Biolabs, Ipswich, MA, USA), assessed with a Bioanalyzer 2100 (Agilent Technologies, Santa Clara, CA, USA), and sequenced using a HiSeq 2000 instrument (Illumina). When processing data, the default program parameters were used unless otherwise stated. Following demultiplexing with CASAVA, the reads were trimmed with bbduk (http://sourceforge.net/projects/bbmap/) and mapped to the annotated cds sequences of strain MG1655 (GCF_000005845.2) using Salmon (8). The resulting files were converted to bam format using SAMtools (9). Transcript abundances were imported in the R environment with tximport (10). Deep sequencing of the global transcriptome yielded 126,950,957 reads in total with an average depth of 10.5 million reads per sample (Table 1). DESeq2 on the R platform was used for calculation of differentially expressed genes (11). The top 2,000 genes ranked by standard deviation were generated using iDEP version 2.0 (12) and used to construct the heat map (Fig. 1).

**TABLE 1:** Summary of sequencing reads^*a*^

Sample	Treatment	No. of clean mapped reads	SRA accession no.	GEO accession no.
K1	Control	10,540,158	SRX17858407	GSM6631702
K2	Control	9,319,226	SRX17858410	GSM6631705
K3	Control	9,149,460	SRX17858413	GSM6631708
Nb1	100 mg/L novobiocin	10,616,875	SRX17858416	GSM6631711
Nb2	100 mg/L novobiocin	10,212,412	SRX17858419	GSM6631714
Nb3	100 mg/L novobiocin	10,016,476	SRX17858422	GSM6631717
Rif1	50 mg/L rifampicin	9,785,875	SRX17858425	GSM6631720
Rif2	50 mg/L rifampicin	10,675,174	SRX17858428	GSM6631723
Rif3	50 mg/L rifampicin	9,588,376	SRX17858431	GSM6631726
Tet1	12.5 mg/L tetracycline	11,029,252	SRX17858434	GSM6631729
Tet2	12.5 mg/L tetracycline	10,427,468	SRX17858437	GSM6631732
Tet3	12.5 mg/L tetracycline	10,379,156	SRX17858440	GSM6631735

^*a*^ The numbers of reads after their filtering and mapping to the reference genome are given. For all libraries, the length of reads was 57 bp, Q > 32 according to the Phred scale.

The gene expression profile shows clear differences in the transcriptome depending on the antibiotic used (Fig. 1). These data will allow a better understanding of global and specific changes in gene regulation in *E. coli* in response to antibiotics of different modes of action.

## Data Availability

The RNA-seq raw reads have been deposited in the NCBI SRA and are accessible through BioProject accession no. PRJNA889731. The DESeq results announced here have been deposited in the NCBI Gene Expression Omnibus (GEO) and are accessible through GEO series accession no. GSE215300. The results of expression profiling can be accessed through the link https://www.ncbi.nlm.nih.gov/geo/query/acc.cgi?acc=GSE215300. These data may be relevant in the context of previously obtained results (1–4) and may be useful for future studies of bacterial resistance mechanisms.
